# DNA barcoding as a tool to monitor the diversity of endangered spring snails in an Austrian National Park

**DOI:** 10.3897/BDJ.11.e91496

**Published:** 2023-01-11

**Authors:** Hannah C Schubert, Michael Duda, Anita Eschner, Erich Weigand, Luise Kruckenhauser

**Affiliations:** 1 Central Research Laboratories, Natural History Museum, Vienna, Austria Central Research Laboratories, Natural History Museum Vienna Austria; 2 Department of Evolutionary Biology, University of Vienna, Vienna, Austria Department of Evolutionary Biology, University of Vienna Vienna Austria; 3 3rd Zoological Department, Natural History Museum Vienna, Vienna, Austria 3rd Zoological Department, Natural History Museum Vienna Vienna Austria; 4 Nationalpark OÖ Kalkalpen Ges.m.b.H., Molln, Austria Nationalpark OÖ Kalkalpen Ges.m.b.H. Molln Austria

**Keywords:** DNA barcoding, COI, spring snails, Hydrobioids, national parks, species delimitation

## Abstract

The Kalkalpen National Park is situated in Upper Austria and contains more than 800 springs. The international importance of this Park is, from the perspective of nature conservation directives, highly significant (European Nature Reserve Natura 2000, recognised wetland of the Ramsar convention). In the current study, the hydrobioid fauna (‘spring snails’) of the Kalkalpen National Park was evaluated. These tiny snails are difficult to determine; however, their investigation is especially desirable, as several species are threatened and as they are important for water quality assessment. Snails collected in 39 selected springs were examined with classical morphological methods (shell and genital anatomy) and, subsequently, by DNA analysis. For this task, the DNA barcode, a partial sequence of the mitochondrial cytochrome c oxidase subunit 1 (COI) gene (length of the sequence 658-682 bp), was PCR amplified and sequenced. From 107 specimens, the DNA barcoding sequence could be obtained and compared with already existing DNA sequences. The (sub)endemic species *Bythinellaconica*, *Hauffeniakerschneri*, *Hauffeniawienerwaldensis* and *Belgrandiellaaulaei* could be clearly identified. For *Bythiospeumnocki*, despite the ambitious collecting effort, only empty shells were found in four springs (including the locus typicus spring) in the Park and its surroundings. The genus *Bythinella* was detected in 36 springs. From 25 of these localities, DNA barcodes could be created, which matches those of *Bythinellaconica* (comparison data from ABOL). It is, therefore, concluded that the species occurs widely in the Kalkalpen National Park. The genus *Hauffenia* was sampled from 16 springs. From one, the haplotype of *Hauffeniawienerwaldensis* could be identified (spring is 5 km outside the Park) and from six, the haplotype of *Hauffeniakerschneri*. *Belgrandiellaaulaei* was found in three springs, which all lie outside the boundaries and are, therefore, not included in the protection measures of the National Park. The data and analyses obtained contribute to the assessment of the taxonomic status of the species studied. The present study gives a good baseline for further monitoring of the hydrobioids in the Kalkalpen National Park, which is important to evaluate current as well as to decide on future protection measures for this group.

## Introduction

Biodiversity - the variety of life on earth - encompasses all living organisms and their diversity. This includes the diversity of species, the diversity within species and the diversity of communities of species. In recent years, a high loss of biodiversity has been recorded, partly caused by humans. Knowledge about the diversity of nature is the key prerequisite for developing strategies to protect it.

Small-scale monitoring of biodiversity includes examining the abundance and distribution of a group of organisms to detect long-term changes. National parks and other protected areas are subject to a reporting obligation on the status of their protected areas and protection measures. To be able to show changes in biodiversity and biodiversity loss, the status quo must be recorded regularly. This process is usually very labour-intensive and only possible with existing taxonomic expertise.

In the current study, monitoring of a selected group of animals, namely the hydrobioids (spring snails) in the Austrian Kalkalpen National Park and its surroundings was performed. Classical monitoring reaches its limits with the small spring snails that are morphologically difficult to determine, which is why the DNA barcoding tool is used here.

### Hydrobioids

Hydrobioid is a non-taxonomic, functional term for the totality of hydrobiid (Hydrobiidae Stimpson, 1865) and hydrobiid-like taxa, first defined by Davis (1979) and later reused by Kabat and Hershler (1993), who subjected the family Hydrobiidae s.l. Troschel, 1857 to a review and Wilke et al. (2013), who have studied the phylogenetic relationships of hydrobioids. The use of this term is necessary because the monophyly of the family Hydrobiidae s.l. was clearly rejected by Wilke et al. (2001) and Wilke et al. (2013). The genera that belonged to this family are now assigned to various other families (for example, *Bythinella* Moquin-Tandon, 1856 to Bythinellidae Locard, 1893, *Belgrandiella* A. J. Wagner, 1928 and *Hauffenia* Pollonera, 1898 to Hydrobiidae and *Bythiospeum* Bourguignat, 1882 to Moitessieriidae Bourguignat, 1864) and are all part of the superfamily Truncatelloidea Gray, 1840. All hydrobioids are very small, 0.5 to 8 (maximum 15) millimetres in size (Miller et al. 2018), gonochoristic freshwater gastropods. Their vernacular name ‘spring snails’ is due to the fact that most of the stygobiont snails live in springs, but also caves and interstitial habitats (Falniowski 2018). They have a low ability for dispersal and a limited distribution range (Miller et al. 2018 (Hydrobiidae s.s.); Strong et al. 2008). Sympatric occurrence of different species of the same genus is rare (Glöer 2002 (Hydrobiidae s.l.); Wilke et al. 2010).

Hydrobioids include the most genera within the freshwater gastropods (Glöer 2002 (Hydrobiidae s.l.)). Over 1000 species are described (Falniowski 2018), Strong et al. (2008) estimating the possible number to be in the order of 8000. The classification of hydrobioids is largely based on shell morphology and distribution (Falniowski 2018).

Several taxa of hydrobioids are morphologically and anatomically highly variable (Wilke et al. 2013). Hence, both delimiting species and assigning individuals to an existing species are very difficult only by morphological methods. Even though Boeters (1999) gives well-developed instructions about the preparations of small Prosobranchia, there are still only few robust anatomical characters, which could be used for determination. Reasons for this are, that, because of their small size, hydrobioids have a reduced morphology and that convergence in anatomical features is common in Rissooidea (Kabat and Hershler 1993 (Hydrobiidae s.l.)). Wilke et al. (2001) note in addition, that intraspecific variation of anatomical characters is very high in hydrobioids (Hydrobiidae s.l.). Already Szarowska and Wilke (2004) showed the necessity to include molecular studies in addition to detailed anatomical studies when it comes to the taxonomic classification of this group of snails (Hydrobiidae s.l.). Delicado and Ramos (2012) pointed out, that for the delimitation of hydrobioids, molecular data would be useful to support morphological analyses (Delicado et al. 2012 (Hydrobiidae s.s.). Falniowski (2018) also confirms the need for molecular data in this context.

Amongst the hydrobioids, many species are endemic (Strong et al. 2008). Miller et al. (2018) found 83% of their 906 studied hydrobioid species (Hydrobiidae s.s.) as endemics. Due to their restricted distribution, they are highly endangered by habitat loss (Miller et al. 2018 (Hydrobiidae s.s.)). One destruction event may be enough to wipe out the only known population of a species (Strong et al. 2008) and, thus, can lead to extinction. Of the 1117 species of hydrobioids (here, the old *sensu lato* definition of Hydrobiidae is still used) listed on the IUCN Red List of Threatened Species (status March 2020), 31 are extinct and 536 (ca. 48%) are at least vulnerable. Miller et al. (2018) predict that there will be a higher risk for hydrobioids (Hydrobiidae s.s.) in the future, because of global climate change and the resulting destruction of ecosystems.

Reischütz and Reischütz (2007) listed 42 hydrobioid species of nine genera for Austria (currently belonging to five different families). Thirty five of them were classified as endemics and three as subendemics. The authors categorise one species as not evaluated, two as data deficient, one as least concern, four as near threatened, one as vulnerable, three as endangered, 28 as critically endangered and four as extinct. Excluding the only invasive hydrobioid species *Potamopyrgusantipodarum* Gray, 1843 (Glöer 2002 (Hydrobiidae s.l.)), all native hydrobioids require uncontaminated to very low contaminated waters (Nesemann and Reischütz 2002 (Hydrobiidae s.l.)). The presence, decline or even absence of these spring inhabitants allows conclusions to be drawn about the quality of the water, which also makes them ideal bioindicators or indicator species (Nesemann and Reischütz 2002, Zulka 2014 (Hydrobiidae s.l.)). Reischütz and Reischütz (2009) criticise the way hydrobioids (Hydrobiidae s.l.) are protected in Austria. One example the authors give, is that the protection of all hydrobioids in general (as is the case in Lower Austria), also includes the invasive species *P.antipodarum* (a harmful organism). Another is that habitats (i.e. springs) continue to be destroyed, amongst others for drinking water production.

### Kalkalpen National Park

This study focuses on the hydrobioids of the Kalkalpen National Park, hereinafter abbreviated as Kalkalpen NP, which is situated in Upper Austria and comprises approx. 209 km². It is of utmost importance from a nature conservation perspective. Established in 1997, the area has been internationally recognised as a National Park (according to IUCN category II) since 1998. Since 2004, the NP is a recognised wetland of the Ramsar Conservation and, also since 2004, part of the European 'Natura 2000' nature reserve network.

The Kalkalpen NP comprises more than 800 springs. These springs reflect the characteristics of their catchment area and indicate environmental changes, human interventions and disturbances of the catchment area. The abundance of springs in the Park is typical for the Karst landscape and gives rise to a variety of spring forms. They can provide a suitable habitat for highly specialised species. The National Park staff has been researching the springs since its beginnings and carries out detailed monitoring of some of them, evaluating physical, chemical and microbiological parameters (Stadler 2017).

The Kalkalpen NP is of particular importance for biodiversity in Austria, in accordance with its function as a "hot spot" for endemics and Red List species, which extends beyond the region (Steger 2012). Steger (2012) lists eight endemics and one subendemic for the National Park area as well as 15 Red List mollusc taxa. The high number of endemics and other gastropods worth being protected (especially in the area of springs and alpine regions of the Park - see Steger 2012) is of great significance for Austria.

The state of knowledge on the occurrence of hydrobioids in the Kalkalpen NP is very incomplete. First surveys yielded two new species for the area, *Belgrandiellaaulaei* Haase, Weigand & Haseke, 2000 and *Bythiospeumnocki* Haase, Weigand & Haseke, 2000 (Haase et al. 2000). These new taxa were studied morphologically and anatomically, respectively. In total, four species or species complexes from the genera *Bythiospeum*, *Belgrandiella*, *Bythinella* and *Hauffenia* were detected in the last years (Haseke and Weigand 2000, Aescht and Bisenberger 2011, Steger 2012, Weigand 2012, Weigand 2016). The occurrence of *Bythinellaaustriaca* (Frauenfeld, 1856), *Bythinellaconica* Clessin, 1910 and *Hauffeniakerschneri* (S. Zimmermann, 1930) (however, two morphotypes could be identified for the genus *Hauffenia*) is discussed (Steger 2012, Weigand 2012). Steger (2012) specifically suggested that the hydrobioids of the NP area should be subjected to a thorough inventory and genetical investigation.

### DNA barcoding as a tool for monitoring

In the year 2003, Hebert et al. (2003) introduced the term DNA barcoding, for using molecular tools for animal species identification. A major argument for DNA barcoding is the great diversity of life and the collapsing taxonomic expertise. Where morphological species identification comes to its limits, for example, with cryptic taxa, morphological variation, phenotypic plasticity, different life stages or small samples of organisms, molecular methods should help. Hence for the monitoring of difficult to determine organisms, DNA barcoding is an excellent tool to improve the data availability, but this is, like most of the other applications, constrained by the existence of exhaustive reference databases for DNA barcodes. Consequently, several international and national initiatives were established with the aim to build up such a reference database, like the ABOL – (Austrian Barcode of Life, www.abol.ac.at) initiative, which aims to record the Austrian biodiversity of animals, plants and fungi in an integrative approach that includes DNA barcoding as a standardised method (Szucsich 2015). The DNA barcoding of Austrian molluscs is one project within the Austrian Barcode of Life initiative (www.abol.ac.at/project/mollusken/), which has been conducted since 2014 at the Natural History Museum Vienna (NHMW).

A total of 81 DNA barcodes of 17 hydrobioid species have already been barcoded within the ABOL Mollusca Project, including the genera *Belgrandiella*, *Bythinella*, *Bythiospeum*, *Graziana* Radoman, 1975, *Hauffenia*, *Potamopyrgus*, *Iglica* A. J. Wagner, 1928 and *Lithoglyphus* C. Pfeiffer, 1828 (Status May 2022). Even though these data are not publicly accessible yet, some of the barcodes will be published in the course of this study.

### Aims

In the initial situation for the present survey of the hydrobioids of the Kalkalpen NP, it was assumed that a high diversity of spring-dwelling snails can be found in this area, which is characterised by the numerous and less dynamic springs of the Reichraminger Hintergebirge (Stadler 2017). Some representatives of the previously listed genera in the Kalkalpen NP could not be clearly identified to species level by morphological-anatomical studies due to partly vague descriptions of characteristics, which refer to minor shell-morphological and anatomical differences (partly also intraspecific variation).

The main aim of the present study is a detailed survey of the hydrobioid taxa in selected springs of the Kalkalpen NP. This is to be achieved by morphological determination, photographic documentation and the creation of DNA barcodes from hydrobioid snails. Above all, endemic species that require special protection should be addressed. Moreover, the genetic distinction of different morphotypes within a genus should be evaluated by DNA barcodes. The generated DNA barcodes are then to be compared with existing reference data (from ABOL and BOLD). In addition, reference DNA barcodes are to be created from newly-acquired genetic data. This study will evaluate not only the status quo of the hydrobioids of the Kalkalpen NP, but will also serve as a model study and facilitate future monitoring of hydrobioids, especially in the Kalkalpen NP and its surroundings.

## Material and methods

### Sampling and specimens processing

The samples were collected from 39 springs of the Kalkalpen NP and its surroundings. The majority of samples were collected between October 2018 and April 2020. Different sampling methods were used: hand-picking, using a fine sieve, scooping with a small container or using a net. Suppl. material 1 lists all localities, from which samples were processed during this study, including their abbreviation and additional information on the sites (location, type of spring, drainage direction). Most of the springs examined were sampled once, in seven cases, two collecting events were evaluated. Collecting was conducted mainly not further than 15 metres from the spring outlet. All in all, the distance varies between 0 to 300 metres.

The samples were delivered frozen in volumes between 100 and 500 ml (together with substratum), then thawed, the specimens picked under a binocular viewer and preserved in 80% ethanol before processing. All together, 58 samples (from 39 different localities) of hydrobioid species were obtained. All specimens are deposited in the Mollusca Collection of the NHMW together with additional material, which was not included in this study (Acqu.Nr. 2019.V.).

Specimens which were selected for molecular analyses and empty shells of the genus *Bythiospeum* were photo-documented from the dorsal and ventral sides including a scale under a Nikon SMZ25 stereomicroscope with a Nikon DS-F2.5 camera. The imaging software NIS Elements Version 5.02 was used to create multifocus images.

### Morphology and anatomical examination

Morphological identification at genus level was performed on the basis of the outer shell and essentially followed Glöer (2002). For critical morphological investigations on species level, selected hydrobioid specimens were examined by M. Haase (University Greifswald, Germany), a well-known specialist who described several Austrian hydrobioids (Haase 1992a, Haase 1992b, Haase et al. 2000). Five putative *B.aulaei* from the OCHS spring and three from the BRUN spring, as well as five supposed *Hauffeniawienerwaldensis* Haase, 1992 from the KREMS spring and five putative *H.kerschneri* from the JÖA spring were confirmed by him.

To clarify the observed morphological variation of *B.conica*, 20 individuals were dissected. The specimens were photographed, then the shell dissolved by placing the snails in 0.5 molar EDTA with a pH of 7.5 for 48 hours. The remaining soft bodies were converted in 80% ethanol (Verhaegen et al. 2018), then again photographed, dissected and finally the genital tract examined following illustrations from Boeters and Knebelsberger (2012) for orientation.

### DNA extraction and COI amplification

As the investigated taxa are very small, the entire organisms were used in the DNA extraction and, thus, depleted during the reaction. Usually, DNA barcodes from three individuals per spring were generated and one reference individual was kept as a paravoucher for the NHMW collection. In the case of fewer individuals per sample, one animal was always kept aside (unless there was only one individual) and DNA was extracted from the remaining.

DNA extraction was performed using Qiagen's DNeasy Blood & Tissue Kit following the associated protocol. Lysis was usually carried out for 2.5 hours, in a few cases overnight. Elution was performed twice, each time with 40 µl of elution buffer. DNA concentration was measured with the Invitrogen Qubit Fluorometer from Thermo Fisher Scientific. The Qubit™ dsDNA HS Assay Kit with the associated standard protocol was used.

As it has been shown that amplification of the barcoding region is often problematic in hydrobioids, we designed a set of new primers, by optimising LCO1490 and HCO2190 (Folmer et al. 1994). In addition, two reverse primers were set outside the 3’-end of the barcoding region (HCO2216_Mol3 and HCO2216_Hyd3). For first implementations, the primer pairs LCO1490_Mol1/HCO2198_Mol1 (Duda et al. 2017/Duda et al. 2017) and LCO1490_Mol1/HCO2216_Mol3 (Duda et al. 2017/5’-CCDGGDARAATYAAAATATA-3’) of the ABOL Mollusca project were used. However, consistently, the best results could be achieved with the primer pair LCO1490_Hyd1/HCO2216_Hyd3 (5’-TCAACAAATCATAAGGAYATTGG-3’/5’-CCGGGGAGAATTAAAATATA-3’) specially redesigned for this study and optimised for the investigated taxa. In one case, the primer pair LCO1490_Hyd1/HCO2198_Hyd1 (5’-TCAACAAATCATAAGGAYATTGG-3’/5’-TAAACTTCTGGGTGTCCAAARAATCA-3’) was used. The QIAGEN Multiplex PCR Kit was used and the associated manufacturer's protocol was followed. In most cases, 1 µl of DNA (variable concentrations between 0.08 and 54 ng/μl) was used for the PCR; in a few cases where the PCR failed and had to be repeated, 3 µl were used. PCR amplification was performed under the following conditions: 95°C for 15 min, 35 cycles of (94°C for 30 s, 48/50°C (primer pair dependent) for 90 s and 72°C for 90 s) and 72°C for 10 min.

The PCR products were checked on a 1% agarose gel and cleaned with the QIAquick PCR Purification Kit (Qiagen).

Bidirectional sequencing was performed by Microsynth Austria GmbH using the PCR primer pairs.

### Data analyses

The sequences (Kalkalpen NP and ABOL Mollusca) were assembled, edited and aligned using Geneious Version 10.2.6 (http://www.geneious.com, Kearse et al. 2012).

All created DNA barcodes and their associated data, like photos, scf files and data spreadsheets (including voucher info, taxonomy, specimen details and collecting data), were uploaded to the Barcode of Life Data system (BOLD) (https://www.boldsystems.org/, Ratnasingham and Hebert 2007). None of the sequences was flagged, which indicated that there were no problematic records. All sequences are “Barcode Compliant”. “The standards include a minimum sequence length of 500 bp, less than 1% ambiguous bases, the presence of two trace files, a minimum of low trace quality status and the presence of a country specification in the record as set out by the Consortium for DNA Barcoding (CBOL)” (Milton et al. 2011).

BOLD was used to check which genera and species already have public DNA barcodes, which were then used for comparison with the DNA barcodes generated in this study (status March 2021). In BOLD, the sequences are assigned to so-called BINs (Barcode Index Number) (Ratnasingham and Hebert 2013), which groups sequences into clusters (operational taxonomic units are generated), depending on their genetic similarity. For more detailed information on how a BIN is formed, see Ratnasingham and Hebert (2013). With the help of the BIN analysis on BOLD, it was possible to determine which DNA barcodes were grouped together and with which other species they share the BIN. In addition, the p-distances within a BIN and between neighboring BINs can be read off. The BOLD numbers and BINs can be found in Suppl. material 2.

For comparison, unpublished DNA barcodes from additional specimens, generated in the course of the NHMW-ABOL Mollusca project, were used: for *Belgrandiellafuchsi* (Boeters, 1970), *Belgrandiellamimula* Haase, 1996, *Belgrandiellaparreyssii* (L. Pfeiffer, 1841), *Belgrandiellawawrai* Haase, 1996, *Bythinellaaustriaca* (Frauenfeld, 1857), *Bythinellaconica* Clessin, 1910 and *Hauffeniawienerwaldensis* Haase, 1992, the data will be published in the course of this study.

Genetic distance estimations were calculated with Mega version 7 (Kumar et al. 2016) using the no variance estimation method, p-distances, uniform rates and pairwise deletion as missing data treatment. Transitions and transversions were included as substitutions.

Nucleotide and haplotype diversities were calculated with DnaSP version 5.10 (Librado and Rozas 2009).

A Minimum Spanning Haplotype Network (Bandelt et al. 1999) with the available sequences of the genus *Belgrandiella* (ABOL project and this study) was created with PopART version 1.7 (Leigh and Bryant 2015).

QGIS version 3.6.1 (QGIS.org 2020) was used to create all figures of maps. Layers of Natural Earth, downloaded from www.naturalearthdata.com (September 2019), of OpenStreetMap, downloaded from download.geofabrik.de and of Umweltbundesamt GmbH - data.umweltbundesamt.at, downloaded from www.data.gv.at (September 2019), were used.

## Results

### Collecting success and photo documentation

During the present study, 39 springs of the Kalkalpen NP were examined; all of these were accommodating at least one genus of hydrobioids. In 35 localities, living individuals were collected and, thus, tissue for molecular analyses was available. Table 1 shows the number of springs, in which the different hydrobioid genera could be found. Suppl. material 1 lists all springs investigated and genera found. In addition, the Table shows detailed information on the collecting events. In 15 localities, more than one genus was found.

The number of shells found in one sample varies between one and more than one hundred, depending on the spring, collecting method and genus. In general, higher numbers could be achieved with a net or a scoop, than by hand collecting. Substantially fewer shells were discovered of the smaller genera *Belgrandiella*, *Bythiospeum* and *Hauffenia*. While for *Bythinella* mainly living specimens were found, for *Hauffenia* more empty shells than shells containing tissue were collected. For the genus *Belgrandiella* only few shells could be found, but these usually contained tissue. Of the genus *Bythiospeum*, with the exception of the spring REUT, only few specimens were located and all collected shells were empty. The number of all collected shells is also shown in Suppl. material 1.

**Figure 1.**
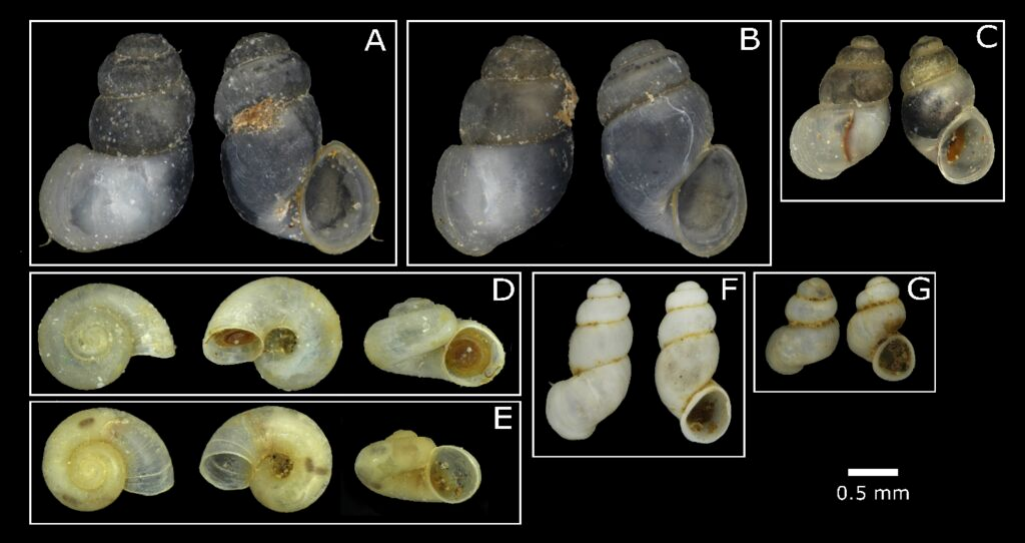
Hydrobioid taxa found in the Kalkalpen National Park. Scale 0.5 mm. **A**: Dorsal (left) and ventral (right) view of *Bythinellaconica*, individual ABOL_510_3 morphotype 1 from the spring KEHLS. **B**: Dorsal (left) and ventral (right) view of *Bythinellaconica* individual ABOL_510_5 morphotype 2 from the spring KEHLS. **C**: Dorsal (left) and ventral (right) view of *Belgrandiellaaulaei* individual ABOL_546_1 from the spring BRUN. **D**: Dorsal, ventral and lateral (left to right) view of *Hauffeniakerschneri* individual ABOL_512_1 from the spring SULZ 2. **E**: Dorsal, ventral and lateral (left to right) view of *Hauffeniawienerwaldensis* individual ABOL_517_1 from the spring KREMS. **F**: Dorsal (left) and ventral (right) view of one individual of Bythiospeumcf.nocki morphotype 2 from the spring REUT. **G**: Dorsal (left) and ventral (right) view of one individual of Bythiospeumcf.nocki morphotype 1 from the spring REUT.

Overall, 343 photos from 164 individuals were taken during this study. One aim was to create a documentation of the specimens, which can be used as a reference, since, for the DNA analysis, the whole animals were used. The photos were uploaded to BOLD along with the DNA barcodes to make them available to the public. Fig. 1 shows example photos from each species, as well as from different morphotypes of *Bythinella* and *Bythiospeum*.

### Morphological identification of species

Based on size, shell shape and the visibility and colour of the operculum, adult snails can be determined at genus level quite well, whereas determination of juvenile hydrobioids often cannot be done unambiguously.

Morphological determination of species is especially difficult in the very small genera *Hauffenia* and *Belgrandiella*. The collected specimens of the genus *Belgrandiella* resembled the species *B.aulaei*, which was described in the Kalkalpen NP. Further confirmation was achieved of eight individuals of two different springs by M. Haase.

At first glance, no clear morphological differences in the individuals of the genus *Hauffenia* were recognisable. However, the molecular analysis revealed two quite different haplotypes of this genus (see below). Hence, five individuals from the spring KREMS and five from the spring JÖA were determined by M. Haase: the specimens from KREMS as *H.wienerwaldensis* and the ones from JÖA as *H.kerschneri*

Shell morphology and the location of the springs, in which the *Bythiospeum* specimens were found, indicated that the collected specimens belong to the species *B.nocki*, with the locus typicus at the spring REUT (Haase et al. 2000). Two different morphotypes were identified: with large differences in size and, sometimes, even in the number of whorls. Besides the smaller morphotype 1 (with an approximate size of 1 mm - no exact measurements were taken in this study) and the large morphotype 2 (with an approximate size of 1.5 mm and larger) also transitional forms occurred. At the locus typicus spring REUT, both morphotypes were detected. Out of 50 shells found there, 35 were assigned to the large morphotype and 15 to the small morphotype.

The morphology of the specimens of the genus *Bythinella* pointed towards the species *B.conica* and *B.austriaca*, which cannot be distinguished by morphological characteristics, but the location data (Boeters and Knebelsberger 2012, Ternus et al. 2019) indicate that the collected individuals belong to *B.conica*, which was supported by the DNA barcodes (see below). In this genus, also two morphotypes have been detected, which were differentiated by their aperture. One of the morphotypes has a clear detached peristome (morphotype 1), while the peristome of the other one fits the shell (morphotype 2) (see Fig. 1). To clarify whether the different morphology corresponds to the sex of the specimens, 10 individuals of each morphotype from the spring KEHLS (here these two phenotypes were particularly noticeable) were dissected. No clear differences in the proportion of one sex was found: for morphotype 1, six males and four females were determined and for morphotype 2, three specimens were determined as females, one as male, the rest as juveniles. Therefore, in both morphotypes, males and females could be found. To evaluate whether the different morphotypes could be different species, DNA barcodes were generated, but no differences were found (see below).

### DNA barcoding success

During this study, DNA was extracted from 111 snails from the Kalkalpen NP and its surroundings. DNA concentrations ranged from 0.08 ng/µl to 54 ng/µl (mean 18.69 ng/µl) for the first eluate and from 0.09 ng/µl to 48.6 ng/µl (mean 8.8 ng/µl) for the second eluate. Some concentrations were too low to measure. The three samples of the genus *Bythiospeum*, which were assumed not to contain tissue, did not yield positive results. A total of 107 DNA barcodes could be generated and all were assessed as barcode compliant (one PCR product failed two times in the sequencing process). According to the quality standards of BOLD, all trace files, except from one specimen (ABOL_532_1), exhibited a high quality. In Table 2, the number of generated DNA barcodes and the number of locations are indicated for each genus.

An overview of all genetically-examined individuals with Sample ID, BOLD numbers and BIN Affiliation can be found in Suppl. material 2. The dataset with all sequences of the Kalkalpen NP, as well as the ones from the project ABOL Mollusca that are used for comparison and published in course of this study, is compiled in BOLD under the code HydHSpub.

### Haplotypes of the Kalkalpen National Park

The DNA barcodes of all species of the Kalkalpen NP showed very low genetic diversity. All measurements can be found in Table 3. Of the 89 DNA barcodes of the genus *Bythinella*, only two were different at one position (each on a different). The 11 DNA barcodes of the genus *Hauffenia* split into two genetically well-differentiated clades, that reflect two species. Within each of the clades, there is no or minimal genetic diversity (Table 3). The haplotypes of individuals of the genus *Belgrandiella* are identical, except for one (which has one substitution).

### Sequence comparison with other Austrian hydrobioids

All generated DNA barcodes were compared with sequences from the genera from the ABOL Mollusca project. A total of 79 hydrobioid sequences from all over Austria (and two individuals from Germany) were available in the ABOL project (not published yet) and form a good comparative database for the species studied here. The sequences of the hydrobioid species which are used for the comparisons below are published on BOLD (Suppl. material 2).

The *Bythinella* sequences match those of *B.conica*, which were collected from Upper Austria, Lower Austria and Salzburg. Compared to the sequences of *B.austriaca*, which has a small genetic distance to *B.conica* and is morphologically indistinguishable (Boeters and Knebelsberger 2012), most of the sequences are separated by six characteristic substitutions. The genetic distances of the DNA barcodes between the two *Bythinella* species range from 0.59% to 0.94% and are higher than within *B.conica* (98 individuals, 0 to 0.29%) and within *B.austriaca* (24 individuals, 0 to 0.16%). *B.austriaca* is known from the East of Austria, while *B.conica* is found in the western regions, the closest occurrence of both species being in the Wildnisgebiet Dürrenstein-Lassingtal (approx. 5.6 km apart) (Fischer and Duda 2019). These findings are concordant with the investigations of Boeters and Knebelsberger (2012) and Ternus et al. (2019). A map with the sample sites of all *B.austriaca* and *B.conica* specimens from this study and from the ABOL Mollusc Project can be found in Suppl. material 3.

For the sequences of the genus *Hauffenia*, one of the haplogroups from the present study matches perfectly with a sequence of an individual of *H.wienerwaldensis* from Vienna, which was analysed within the project ABOL Mollusca.

**Figure 2.**
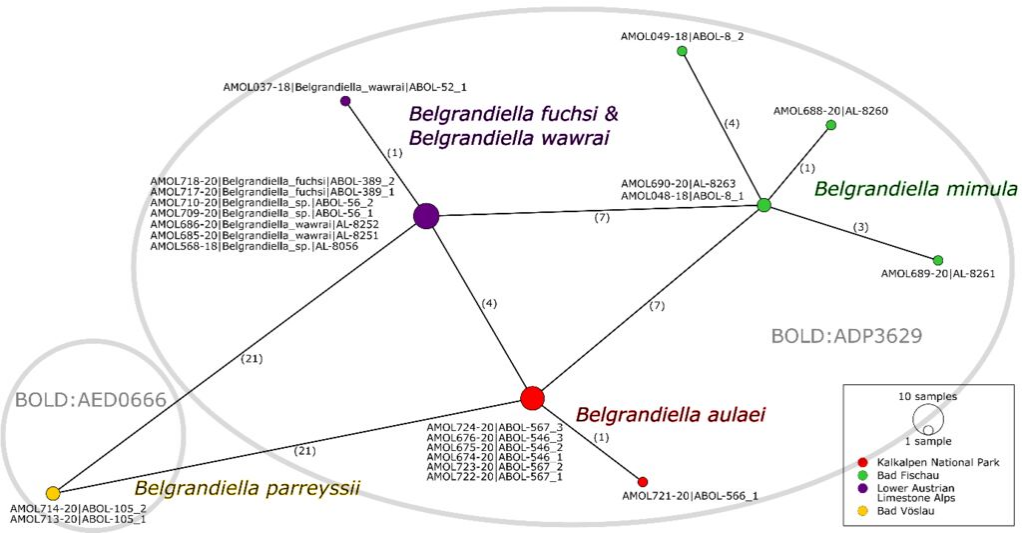
Haplotype Network of all *Belgrandiella* sequences from the present study and from the ABOL Mollusca project. The numbers on the connection lines represent the number of substitutions between the two haplotypes. The different colours indicate different areas, the two grey circles different BINs.

**Figure 3.**
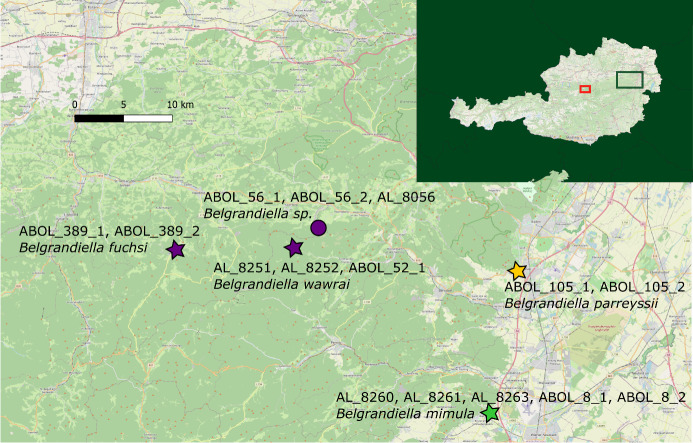
Sample sites of the specimens of the genus *Belgrandiella* from the project ABOL Mollusca, which were used for analysis. The green marks represent individuals from Bad Fischau, the violet marks represent individuals from the Lower Austrian limestone alps and the yellow marks represent individuals from Bad Vöslau. The stars indicate that the location is close to the locus typicus. The green square on the Austria map, located in the upper right corner, indicates the approximate area where the collecting sites are located, the red square indicating the position of the Kalkalpen National Park.

The sequences of *B.aulaei* from the Kalkalpen NP and its surroundings are most similar to some individuals of *Belgrandiellafuchsi* and *Belgrandiellawawrai* from Lower Austria, but do not match exactly. In order to make a more precise statement about the comparison of the different haplotypes, a haplotype network of all sequences of *Belgrandiella*, that were generated in both projects was created (Fig. 2). The sequences of the individuals from the Kalkalpen NP are separated by four substitutions from the sequences to seven individuals that were sampled in Kleinzell, Triestingtal, Lilienfeld and Höfnergraben in the Lower Austrian Limestone Alps. Those individuals were determined as *B.fuchsi*, *B.wawrai* and *Belgrandiella* sp. The sequences of two individuals from Bad Fischau in Lower Austria, which were determined as *Belgrandiellamimula*, are separated by seven substitutions. The greatest distance of 21 substitutions is to two individuals from Bad Vöslau, determined as *Belgrandiellaparreyssii*. The latter also appear in a different BIN on BOLD, which is described below in the next chapter in more detail. The map in Fig. 3 shows the different sample sites of the *Belgrandiella* sequences, that were available from the ABOL Mollusca project and were used for analysis.

### BOLD analysis and Barcode Index Numbers (BINs)

The 107 generated DNA barcodes from the Kalkalpen NP samples were uploaded to BOLD (Barcode of Life Data System) (Ratnasingham and Hebert 2007), which provides some features for analysis and automatically assigns a BIN (Barcode Index Number), based on sequence similarity to existing sequences in BOLD (Ratnasingham and Hebert 2013). A list of all uploaded specimens with their corresponding BOLD number and BIN affiliation is to be found in Suppl. material 2. The genetic distances within the BINs of the Kalkalpen NP representatives and the genetic distances to the Nearest Neighbor BINs are listed in Table 4.

All 89 DNA barcodes of *B.conica* from the Kalkalpen NP were assigned to the BIN BOLD:AAA4467 (see Suppl. material 2). The published records of the BIN also contain taxa that have been identified as other species of the genus *Bythinella*: *B.austriaca*, *B.cylindrica* and *Bythinellametarubra* Falniowski, 1987, as well as undefined *Bythinella*. The individuals are from: Poland (37), Germany (18), Austria (17), Slovakia (16), Hungary (15), Czech Republic (4) and Unknown (2).

The BIN analysis in BOLD also revealed two different BINs within the generated sequences of genus *Hauffenia*. Apart from the sequences from the Kalkalpen NP and the *H.wienerwaldensis* sequence from ABOL, no further sequences are included in the BIN BOLD:ADP3094. The *H.kerschneri* representatives of the Kalkalpen NP are assigned to BIN BOLD:AEC8473, which does not contain other sequences. No other *H.kerschneri* sequences are deposited in BOLD. The BINs are the Nearest Neighbor BIN of each other. The BIN that includes the *H.wienerwaldensis* sequences is also the Nearest Neighbor BIN (BOLD:ADP3094) to two individuals from Slovakia with a distance of 9.03% (BIN BOLD:AAY2140).

The DNA barcodes of *Belgrandiella* from the Kalkalpen NP and its surroundings are assigned to the BIN BOLD:ADP3629. This BIN includes 13 further DNA barcodes from *B.mimula* (5), *B.wawrai* (3), *B.fuchsi* (2) and *Belgrandiella* sp. (3), which all came from the ABOL Mollusca project. The Nearest Neighbor BIN consists of the two DNA barcodes from *B.parreyssii* from Bad Vöslau, collected in the course of the ABOL Mollusca project. There are no other sequences of *B.aulaei* in BOLD for comparison.

### Distribution of Hydrobioids in the Kalkalpen National Park

A full record of the 39 investigated springs of the Kalkalpen NP and all hydrobioids found is listed in Suppl. material 1.

Suppl. material 4 shows the sample sites of hydrobioids in the Kalkalpen NP and its surroundings. The genus *Bythinella* was found in 36 springs all over the Kalkalpen NP and its surroundings. DNA examinations were performed on individuals from 25 springs, which were consistent with *B.conica*. *Hauffenia* was detected in 16 springs of the National Park and its surroundings. In the spring KREMS, *H.wienerwaldensis* occurs, which can be found in the western surroundings of the Park about 5 km outside the border. In six springs (HRQ-E1, HRQ-E2, JÖA, SULZ_B, VRQ and Welchau1+2), *H.kerschneri* occurred. Thus, a distribution in the northwest of the Park can be established. In the south-eastern areas so far, no specimen of the genus *Hauffenia* was detected. In three springs outside the Kalkalpen NP, *B.aulaei* was found. The springs are located in the north, northeast and east outside the borders and are several kilometres apart from each other. *Bythiospeum* was found in four springs in the central north of the Kalkalpen NP and its surroundings. Only empty shells of the genus were found.

## Discussion

### Methodological issues

Living aquatic snails with an operculum tend to retract and seal their shell with the operculum, which prevents the penetration of alcohol into the tissue and could hinder proper fixation and conservation of the tissue. Consequently, this could cause the degradation of genomic DNA and amplification of the whole DNA barcoding fragment would be difficult. Nevertheless, the DNA extraction, PCR and sequencing of the specimens fixed in 80% EtOH worked very well. One reason for this could be that, due to the small size of the snails, the alcohol can still ingress. DNA barcoding of molluscs might raise difficulties, as their high divergence within COI sequences may result in mutations in the primer binding region in multiple taxa and, hence, require adjustments to molecular methods, such as primer design (Kruckenhauser et al. 2019). Additionally, in this study, the primers of the ABOL Mollusca project were adapted for the hydrobioid group. The adapted forward primer LCO1490_Hyd1, as well as the reverse primer HCO2198_Hyd1, contains one wobble. The reverse primer HCO2216_Hyd3 does not contain wobbles and lies 24 base pairs outside the classical Folmer region (Folmer et al. 1994). The binding of the primers worked well in all analysed taxa, as shown by the high DNA barcoding success despite the low quantity of DNA. This shows that the effort of designing specific primers for difficult groups is time- as well as cost-efficient and worthwhile, since laborious replications can be avoided. The longer fragment amplified with the reverse primer HCO2216_Hyd3 also facilitates the design of new primer that spans through the whole Folmer region.

Since none of the sequences was flagged as problematic by BOLD and they all are “Barcode Compliant” (see chapter Material and Methods), the quality of the DNA barcodes can be assumed to be high. In their "The seven deadly sins of DNA barcoding", Collins and Cruickshank (2013) plead for great care in the a priori determination of species, when adding to reference libraries, such as BOLD, as these usually do not carry out any verification of identifications. Therefore, in the current study, a great effort was invested to assure correct determination of the specimens, so the uploaded data should serve as good reference DNA barcodes for these species.

### Species determination and delimitation

The discussion about when a species is a species has been going on for a very long time. There have been many controversies about species concepts and different ways of species delimitation. Since the hydrobioids are not a group that can be easily distinguished morphologically, due to few and diverse characteristics (see Introduction), some studies have been made to delimit hydrobioid taxa using genetic data (see also Introduction). Wilke et al. (2001) investigated the monophyly of hydrobioids and their phylogenetic relationships in 2001. In their study, they chose a combination of the phylogenetic markers COI and a fragment of the nuclear 18S gene and found that both fragments show good performance for this purpose. The COI gene fragment has a consistent performance at the genus and family level, while the 18S gene fragment has a good phylogenetic informative value on and above the family level (Wilke et al. 2001). In the following two decades, several phylogenetic analyses on hydrobioids were carried out using COI (for some examples, see Haase et al. 2007, Benke et al. 2009, Šteffek et al. 2011, Boeters and Knebelsberger 2012, Delicado and Ramos 2012, Falniowski et al. 2012, Richling et al. 2016, Delicado 2018) or a combination of COI with one or more other markers (for some examples, see Bichain et al. 2007b, Falniowski et al. 2009a, Falniowski et al. 2009b, Wilke et al. 2013, Osikowski et al. 2015, Szarowska et al. 2016a, Szarowska et al. 2016b, Delicado et al. 2018, Hofman et al. 2018, Delicado et al. 2019). Wilke et al. (2013), who examined the group of hydrobioids phylogenetically again in 2013, state that a “combination of ‘standard’ gastropod genes is very useful for phylogenetic studies targeting family-groups or lower rissooidean taxa”. Richling et al. (2016) called COI “a suitable marker for first phylogenetic reconstructions” when studying the genus *Bythiospeum* in Europe in 2016. Delicado (2018), investigating the genus *Sadleriana* Clessin, 1890, notes that the COI fragment “provides sufficient resolution to detect intra- and interspecific variation in springsnails”. Bichain et al. (2007), who investigated the species delimitation within the genus *Bythinella*, come to the conclusion that the mitochondrial DNA barcoding gene COI alone is not sufficient to identify a species boundary and that the inclusion of other markers is necessary. Haase et al. (2007) advocate especially an integrative approach with morphological, anatomical and genetic studies for species delimitation in the genus *Bythinella*.

In this study, DNA barcoding was mainly used to compare the generated data with existing data (assign unknown specimens to species) and to create (new) genetic references. For the species *B.aulaei* and *H.kerschneri*, the specimens, which were used to establish the reference DNA barcodes, were determined morphologically and anatomically, which will make it easier to identify these species in the future. For the delimitation of some species, however, the tool of DNA barcoding alone was not sufficient. In order to be able to investigate the differentiation of *B.conica* from *B.austriaca*, as well as *B.aulaei* from other closely-related species of the genus, additional investigations with further nuclear markers would be necessary. The assignment of the species of this study is evaluated below and also discussed in terms of their species status. However, no definitive statements are made about delimitations of individual species.

One of the collecting sites of *B.aulaei* is not far from the locus typicus of the species (approx. 7 km). In addition to this, the specimens were examined by the first author M. Haase himself. These two points and the fact that the generated sequences are almost identical (one different at one position), suggest that the collected snails of this genus represent one species and can be clearly assigned to the *B.aulaei*. An additional analysis of specimens from the locus typicus would complete the picture. For the delimitation of the species to other species of the genus *Belgrandiella* in Austria, based on genetic data, the situation has to be discussed in more detail (the morphological delineations can be found in Haase 1994, Haase 1996 and Haase et al. 2000). In general, it is striking that the intra- and interspecific genetic distances of the Austrian *Belgrandiella* species considered in this study are quite low. The haplotype network in Fig. 2 shows this particularly well. Only one substitution in the approx. 670 bp COI fragment of one of seven specimens shows the intraspecific variance (average distance of 0.04%) of *B.aulaei* here. The minimum substitutions to representatives of other species are four (*B.wawrai* and *B.fuchsi*) and seven (*B.mimula*). The largest distance with 21 substitutions is to *B.parreyssii*. These short distances are one reason why all these species, except *B.parreyssii*, share a BIN in BOLD. The average distance within this BIN, which can be considered as an OTU (operational taxonomic unit), is 0.9%, the maximum distance is 2.03%. The distance to the Nearest Neighbor BIN, which includes exclusively *B.parreyssii*, is 3.29%. Various values for the average genetic divergence of the COI gene amongst species of the family Hydrobiidae can be found in the literature. In their studies on *Pseudamnicola* Paulucci, 1878 species, Delicado et al. (2012) found a mean difference of about 8%. They also list other values of interspecific differences for the family Hydrobiidae from literature: “*Hydrobia* in Wilke, Rolán and Davis 2000, 3-5.5% and *Floridobia*, 0.5-6.1%, *Marstonia*, 1.0-8.5% and *Pyrgulopsis*, 2.8-11.2% in Hershler et al. 2003”. In 2018, Delicado (2018) studied the species of genus *Sadleriana* Clessin, 1890 and found overall average intraspecific differences of 1.8% in the COI fragment. With Liu et al. (2015), who genetically examined the species *Pyrgulopsiskolobensis* (D. W. Taylor, 1987) in 2015, the mean intraspecific divergence was between 0.3% and 2.9%. The inclusion of literature data on the Hydrobiidae family is useful for the discussion of the species status of *B.aulaei*, but it would be unreasonable to derive a general threshold. Falniowski (2018) also recognised that there is no universal rule and that the level of interspecific distances varies amongst different genera. The examples mentioned above show that very small interspecific genetic distances do also occur in other members of the family Hydrobiidae and suggest that the data on *B.aulaei* do not contradict their species status; however, to clearly verify their species status, a larger dataset with more samples, as well as an investigation with nuclear markers, would be necessary. The clear delimitation of *B.parreyssii* is supported, on the one hand because of the separation in the BIN system, on the other hand because of greater genetic distance with similar spatial distance to the other species studied (see Fig. 2 and Fig. 3). Haase (1994) found in his genetic analyses on the basis of allozyme electrophoresis high interspecific distances between *B.fuchsi* and *B.parreyssii*, which is consistent with the data in this study. For *B.wawrai* and *B.fuchsi*, no genetic differences were found in the analysis of the present study. In the haplotype network, they occur in the same haplogroup - their generated COI sequences are identical (except for one with one substitution). Anatomically, however, the two species are separated by the location of the bursa copulatrix (Haase 1996). One reason for the low genetic distances of the *Belgrandiella* species could be that they are relatively young species that might have formed during the Pleistocene. During the Ice Ages, many areas of the Eastern Alps were glaciated, but especially in the north-eastern and south-eastern parts, the ice sheet was never completely closed and could have served as Ice Age refugia for these snails, as was documented for several land snails (Harl et al. 2014, Kruckenhauser et al. 2014, Kruckenhauser et al. 2017). Postglacial recolonisation in Central Europe is also assumed for the hydrobioid genus *Bythiospeum* (Richling et al. 2016). In contrast, Haase (1996) suspects that the Austrian *Belgrandiella* species are rather old, which is not supported by our data. The only exception is *B.parreyssii*, with a genetic distance of approx. 3.29% to the other species, which might have survived the glaciations in a separate refugium. However, given the small sample size and range and the lack of fossil data, it appears not justified to carry out a molecular clock analysis.

The DNA Barcodes of the different morphotypes of *Bythinella* that were collected in the Kalkalpen NP, were identical and, hence, give no indication that these morphotypes represent different species. It is known that the intraspecific and interspecific variability of the shell morphology of the genus *Bythinella* can lead to misidentifications (Glöer 2002). The most likely assumption seems to be that the differences are due to various environmental influences. As early as 1979, the shell variability of the genus was described as ecophenotypic (Falniowski 1987). Falniowski (2018) also points out that “[…] in springs, reproduction takes place throughout a year, but the conditions - like amount of food (e.g. algae) varies between summer and winter, which often results in generations strikingly different in morphology at the same spring, which mimics distinct species”. Another hypothesis was that the different morphotypes reflect sexual dimorphism; this could be rejected by the anatomical examinations in this study. The distribution (Boeters and Knebelsberger 2012, Ternus et al. 2019), as well as the genetic data of the *Bythinella* specimens, clearly assign them to a group of individuals designated as *B.conica*. However, the discussion remains whether this species should be delimited as a separate species or better regarded as a subspecies of *B.austriaca*, as it is suggested, for example, by Glöer (2002). Boeters and Knebelsberger (2012) even divide the species *B.conica* into two subspecies, one of which is geographically isolated in a small area of the Tiroler Ache and is morphologically distinct. The discussion in the present study refers only to the species level. The most important argument to treat *B.conica* and *B.austriaca* as one species is that they do not differ morphologically and anatomically, so the only differentiation is geographical and genetic (Boeters and Knebelsberger 2012). An integrative approach as is suggested by Schlick-Steiner et al. (2010), in general and by Haase et al. (2007) and Bichain et al. (2007a), in particular for the genus *Bythinella*, cannot be adhered to. A study on morphometric differences is currently being conducted at the University of Salzburg and may contribute to new insights into external differences of *B.austriaca* and *B.conica* (Ternus et al. 2019). Already in Boeters and Knebelsberger (2012), a clear genetic distinction in the COI gene between *B.austriaca* and *B.conica* has been described. Although the distances were quite low, a distinct gap between the highest intraspecific distances of 0.43% and 0.87% (mean 0.22% and 0.11%) and the lowest interspecific distances of 1.3% (mean 1.5%) was found. The same pattern can be found in the current study, where the mean interspecific distance between the two groups is 0.88%, which is rather low and let the species on BOLD be assigned to the same BIN. This value is below the threshold of 1.5%, suggested by Bichain et al. (2007) for the delimitation of *Bythinella* species (whereas also much higher intraspecific genetic distances have been found for the genus *Bythinella*, see for example Fehér et al. (2013). Additionally, the mean intraspecific distances were lower in the present study (*B.conica* 0.01%, *B.austriaca* 0.02%). These differences in the distances between the two works can be explained by the fact that the sequences used by Boeters and Knebelsberger (2012) generally have a higher variability. Boeters and Knebelsberger (2012) postulated also that the species *B.austriaca* is more likely to be distributed in the east of Austria and the species *B.conica* in the west. The data obtained here support this hypothesis (see Suppl. material 3). Like Boeters and Knebelsberger (2012), unfortunately no assumptions can be made about the geographical barrier in the present study, even though the nearest collecting sites of the different haplogroups are only about approx. 5.6 km apart. The available data do not indicate that isolation by distance is present, as no increasing difference in divergence with increasing geographical distance can be detected. If *B.conica* and *B.austriaca* are indeed separate species, they could be quite young species that have emerged in the late Pleistocene and, until now, no major genetic differences have formed (Benke et al. (2011) also suspect genetic bottlenecks during the recolonisation after the Pleistocene Ice Ages as a possible cause for the low diversity of the genus *Bythinella* in northern and central Europe). For the genus *Bythinella*, Wilke et al. (2010) found evidence of non-adaptive radiation. In such cases, a morphostatic evolution can be recognised, as is also discussed by Falniowski (2018) for hydrobioids, in which species arise that do not differ from each other either morphologically or ecologically. The question remains whether these are then different species and how distinct the genetic differences would then have to be. The aim of the current study was not to give a definitive answer whether *B.austriaca* and *B.conica* are to be regarded as separate species. However, data were provided that will be helpful for the discussion about it and it could be shown that DNA barcoding provides a good possibility to distinguish the two taxa.

In the course of this study, shells of the genus *Bythiospeum* were (re)found at the locus typicus of *B.nocki* (spring REUT), which was described there in 2000 by Haase et al. (2000) on the basis of shell morphology. This allows the conclusion that at least the snails collected there belong to *B.nocki*. Based on the distribution, the species is also assumed for the remaining findings. One of the localities is also listed as additional material in the first description (spring Welchau) and the other springs SULZ and JOEA are within the radius of about 5 km around the locus typicus, as the additional localities in the first description. Future anatomical and genetic study of living material (which unfortunately was not available in the present study) is inevitable to verify this assumption. Even though spring JOEA belongs to a different catchment area from the other springs, past experience has shown that assuming a new species of the genus *Bythiospeum* due to a different distribution alone can lead to an overestimation of the number of species (Richling et al. 2016). The assessment of the different morphotypes found is difficult in this case, due to the lack of anatomical and genetic data. It cannot be excluded that these are different species, sex differences or differences in development. Differences in generations, as noted in the genus *Bythinella* (see above), could also be a reason for the different morphotypes.

For the genus *Hauffenia*, two genetically well-differentiated clades in the Kalkalpen NP and its surroundings can be newly presented in this study. Until now, no distribution of any species other than *H.kerschneri* was listed in literature for this region and also no morphological differences were identifiable in the first inspection of the specimens. The mean distance between the two clades is 8.08% (mean intraspecific distances are 0.03% and 0.1%) and, thus, also lies in the spectrum of the interspecific distances calculated by Rysiewska et al. (2017) for COI in the genus *Hauffenia*. In BOLD, too, the two clades are assigned to different BINs. M. Haase examined the specimens sent to him (five each) and identified them as *H.wienerwaldensis* and *H.kerschneri*. For the DNA barcodes generated for the *H.wienerwaldensis* group, there was also a match to one *H.wienerwaldensis* sequence available through ABOL. Which of the two subspecies of *H.kerschneri* presented by Haase (1992a) in 1992 occurs in the Kalkalpen NP cannot be answered in the present study; this would require a detailed anatomical examination. The reference DNA barcode created for the species *H.kerschneri*, as well as the additional genetic data for the species *H.wienerwaldensis*, of which only one COI sequence was available so far, will be helpful in the future to assign specimens of *Hauffenia* to a species without difficult anatomical examinations.

### Distribution and ecology

It can be concluded that *B.conica* occurs widely in the Kalkalpen NP, because specimens of the genus *Bythinella* were barcoded from various springs throughout the area and all the sequences generated refer to this species. The locations where the species was found include different catchment areas (areas can be looked up in Stadler 2017). The same applies to *H.kerschneri*, which was mainly collected in the northwest of the Park. To clarify whether the species also occurs in the southeast, further data are required. The presumed species *B.nocki*, which was found in the central north of the National Park, also occurs in at least two different catchment areas (just two sample sites within the Park). Despite the high collecting efforts for *B.aulaei*, only sites outside the Kalkalpen NP could be identified. Further studies are necessary to clarify whether a distribution within the Park is probable. The same applies to *H.wienerwaldensis*, which was only identified in one spring outside the Park. For *B.aulaei*, *B.conica*, *B.nocki* and *H.kerschneri*, new localities within the known overall distribution area (Haase 1992a, Haase et al. 2000, Reischütz 2010a, Boeters and Knebelsberger 2012) can be confirmed. For *H.wienerwaldensis*, the known distribution range (Reischütz 2010b) could be extended with this study.

Apart from the fact that hydrobioids are habitat specialists (Miller et al. 2018) that require uncontaminated water with relatively low temperatures (Moog 2002, Wilke et al. 2010), little is known about the ecology of the group (Falniowski 2018). Wilke et al. (2010) wrote about the genus *Bythinella* that they occur most frequently in rheocrene springs, which is also supported by this study (see Suppl. material 1). Exact comparisons of the individual parameters (amount of water, temperature, pH value, oxygen level, ion concentrations, turbidity, microbes etc.) from the monitoring studies of the Kalkalpen NP with the detected spring snails should be considered in further studies.

### Endangerment and conservation

In his prioritisation of Austrian animal species and habitats for nature conservation measures, Zulka (2014) lists 48 mollusc taxa in the highest prioritisation category for Austria. He therefore recommends, in close connection with the biodiversity concept according to Wilson and Peter (1988), to give endangered species a high priority in nature conservation. Accordingly, all measures regulated by national parks and nature conservation laws, as well as by other official regulations, such as research and monitoring, protection and management plans, must be adhered to and implemented. In general, there is a high number of endemics amongst native snails (Reischütz and Reischütz 2007, Reischütz and Reischütz 2009). In Austria, especially the northern and southern Eastern Alps are a hot spot of endemic invertebrates and vascular plants (Rabitsch and Essl 2009). The reasons for this are the special biogeographical conditions, which are linked, amongst other things, to the history of the Ice Ages. During the Ice Ages, many areas of the Eastern Alps were glaciated, but especially in the north-eastern and south-eastern parts, the ice sheet was never completely closed. Mountain ranges with several altitudinal levels, vertically continuous areas such as rock and debris areas, as well as groundwater systems, offer a potential basis for the formation of endemics. Local survival under adverse climatic conditions is given by the possibility to migrate up-slope during warm phases and again downhill when it cools down. The groundwater systems of the unglaciated mountain ranges in the Eastern Alps also offered long-term stable temperature conditions. Accordingly, numerous endemics are found in hydrobioids (35 of the 42 species currently documented for Austria (Reischütz and Reischütz 2007). With its complex groundwater system (Stadler 2017) and the numerous unobstructed springs, the Kalkalpen NP is of supra-regional importance for this group of animals. Especially the groundwater snails of the genera *Hauffenia* and *Bythiospeum*, living in the crevice system of the Karst, are still insufficiently researched and highly threatened by human impacts or lowering of the groundwater level.

All species investigated in this study are endemics or subendemics (in the case of *B.conica*) (Reischütz and Reischütz 2007) and require special protection. Their restricted habitat and low ability to disperse (Miller et al. 2018) put the spring-dwelling snails at additional risk, as contamination of the spring could wipe out the entire population (Strong et al. 2008, Zulka 2014). Karst systems in particular (around three quarters of the Kalkalpen NP are karstified) are often in direct contact with the surface, so that small amounts of soil contamination are carried to the spring by aquifers (Stadler 2017). Strong et al. (2008) list a variety of potential risks of hydrobioids, including “[…] depletion of ground water for a number of urban and rural uses including water capture for stock, irrigation or mining, spring or landscape modification and trampling by cattle […]” as well as “[…] gravel mining and other sources of mine waste pollution, dredging, channelization, siltation from agriculture and logging, pesticide and heavy metal loading, organic pollution, acidification, salination, waterborne disease control, urban and agricultural development, unsustainable water extraction for irrigation, stock and urban use, competition and/or smothering from introduced species […]”.

Although the special endangerment of the hydrobioids within the Kalkalpen NP has already been recognised before (Jaksch and Steger 2014), this study has contributed significantly to the knowledge of these endemics in the Park. This helps to determine the localities that need to be specially protected and can act as a model study for future monitoring projects. It also provides an opportunity to review existing protection measures and discuss updates. By prioritising its conservation goods, the Kalkalpen NP determines which species and habitat types require special protection (Nationalpark O.ö. Kalkalpen Ges.m.b.H 2018). The protection of springs from grazing cattle is fulfilled, for example, through fences (annual assembly and disassembly necessary) and watering places for the cattle (Nationalpark O.ö. Kalkalpen Ges.m.b.H 2018, Weigand 2008). In the future, regular, long-term monitoring should be considered in order to evaluate the changes in the various springs in the coming years. More in-depth studies, especially on the genera *Hauffenia*, *Belgrandiella* and *Bythiospeum*, would be highly recommended in any case. In addition, an extension of the area of the Kalkalpen NP to the sensitive marginal areas worthy of protection should be proposed to protect *B.aulaei*, which is so far only known from the surroundings of the National Park. If no area extensions are possible, other protection of individual springs outside the National Park should be implemented (e.g. as natural monuments).

## Supplementary Material

6DEB3581-A5D4-56D2-B6D7-D0DA16D5964E 10.3897/BDJ.11.e91496.suppl1 7533443 Supplementary material 1 Supporting Table 1 Data type Table Brief description Springs of the Kalkalpen National Park including information about taxa of hydrobioids, locality details, collecting events and number generated DNA barcodes. File: oo_718833.xlsx https://binary.pensoft.net/file/718833 Hannah Schubert, Michael Duda, Anita Eschner, Erich Weigand, Luise Kruckenhauser

E9E4E5FA-5121-5AB1-BD9D-377DB28D6C68 10.3897/BDJ.11.e91496.suppl2 7533445 Supplementary material 2 Supporting Table 2 Data type Table Brief description Specimens overview of hydrobioid sequences from the Kalkalpen National Park and its surrounding, as well as other Austrian individuals that were used for comparison (indicated by an asterisk): BOLD ID, BIN Affiliation and species name. File: oo_718834.xlsx https://binary.pensoft.net/file/718834 Hannah Schubert, Michael Duda, Anita Eschner, Erich Weigand, Luise Kruckenhauser

BF1B208C-EFBE-52D9-A32E-9A40540FEE6E 10.3897/BDJ.11.e91496.suppl3 7533451 Supplementary material 3 Supporting Figure 1 Data type Figure Brief description Sample sites of the specimens of *Bythinellaaustriaca* (red dots) and *Bythinellaconica* (green dots), which were used for distance analysis. All collected as a part of the projects ABOL Mollusca and hydrobioids of the Kalkalpen National Park (this study). The light green dots represent sample sites of the Kalkalpen National Park project, from where no DNA barcodes were generated, but based on their sampling locality, they are assumed to be *Bythinellaconica*. File: oo_745132.png https://binary.pensoft.net/file/745132 Hannah Schubert, Michael Duda, Anita Eschner, Erich Weigand, Luise Kruckenhauser

9C08EE3A-60C3-5875-A162-F0CC09D52618 10.3897/BDJ.11.e91496.suppl4 7533453 Supplementary material 4 Supporting Figure 2 Data type Figure Brief description Map of the Kalkalpen National Park and its surroundings with indication of occurrence of hydrobioid species. Where stars instead of dots are plotted, also DNA barcodes were generated. A: The blue markings indicate *Bythinellaconica* found in the area. B: The red/white markings indicate the genus *Hauffenia* found in the area. Red stars indicate *Hauffeniakerschneri*, white stars indicate *Hauffeniawienerwaldensis*. C: The yellow stars indicate *Belgrandiellaaulaei* found in the area. D: The green dots indicate the genus *Bythiospeum* found in the area. File: oo_718837.png https://binary.pensoft.net/file/718837 Hannah Schubert, Michael Duda, Anita Eschner, Erich Weigand, Luise Kruckenhauser

## Figures and Tables

**Table 1.** Number of springs, in which hydrobioid genera are found.

	*Bythinella*	*Hauffenia*	*Belgrandiella*	*Bythiospeum*
total	36	16	3	4
alive	35	8	3	-
empty shells	1	8	-	4

**Table 2.** Number of generated DNA barcodes and number of locations per genus.

Genus	generated DNA barcodes	number of locations
*Bythinella*	89	26
*Hauffenia*	11	7
*Belgrandiella*	7	3

**Table 3.** Measures of genetic diversity of the haplotypes of each hydrobioid species of the Kalkalpen NP.

	*Bythinellaconica* (n = 89)	*Hauffeniawienerwaldensis* (n = 3)	*Hauffeniakerschneri* (n = 7)	between *H.wienerwaldensis*/*H.kerschneri*	*Belgrandiellaaulaei* (n = 7)
mean p-distance [%]	0.007	0.1	0.03	8.08	0.04
max. p-distance [%]	0.29	0.15	0.15	8.51	0.15
min. p-distance [%]	0	0	0	7.88	0
haplotype diversity	0.05	0.67	0	n.d.	0.29
nucleotide diversity	0.00007	0.001	0	n.d.	0.0004

**Table 4.** Genetic distances of the BINs, that includes the sequences of the Kalkalpen NP individuals, *new BINs in BOLD

BIN (Barcode Index Number)	BOLD:AAA4467 (including *B.conica* and *B.austriaca*)	BOLD:ADP3094* (*H.wienerwaldensis*)	BOLD:AEC8473* (*H.kerschneri*)	BOLD:ADP3629* (including *B.aulaei*, *B.fuchsi*, *B.mimula*, *B.wawrai*)
mean distance [%]	0.5	0.16	0.03	0.9
max distance [%]	2.61	0.33	0.16	2.03
distance to Nearest Neighbor BIN [%]	4.4	8.05	8.05	3.29
