# Supplementary figures and images for: DNA barcoding as a tool to monitor the diversity of endangered spring snails in an Austrian National Park
Source: Biodivers Data J. 2023 Jan 11;11:e91496. doi: 10.3897/BDJ.11.e91496 (PMC9850253; doi:10.3897/BDJ.11.e91496)

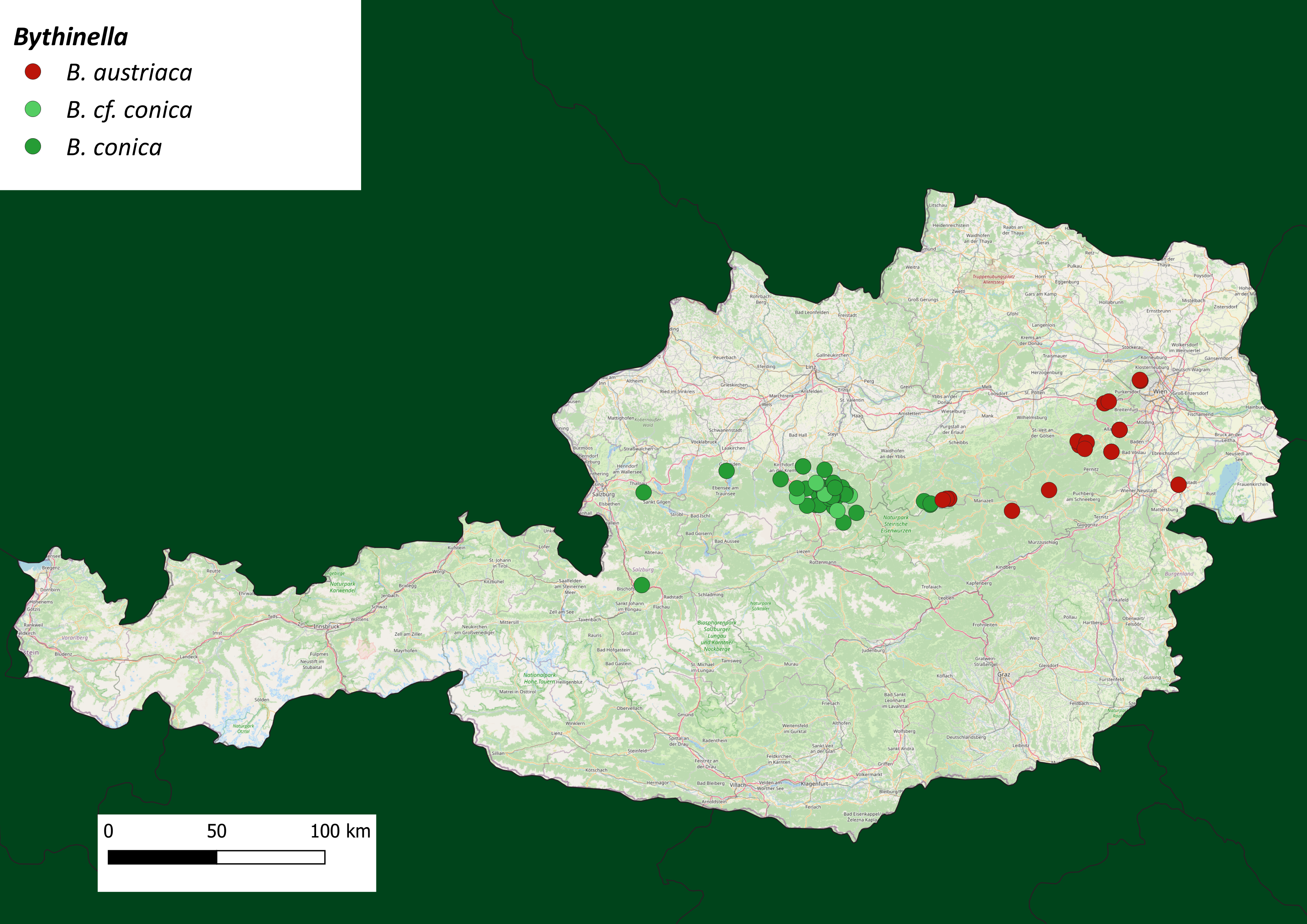

Supplement: Supplementary material 3 — Supporting Figure 1 [file bdj-11-e91496-s003.png]

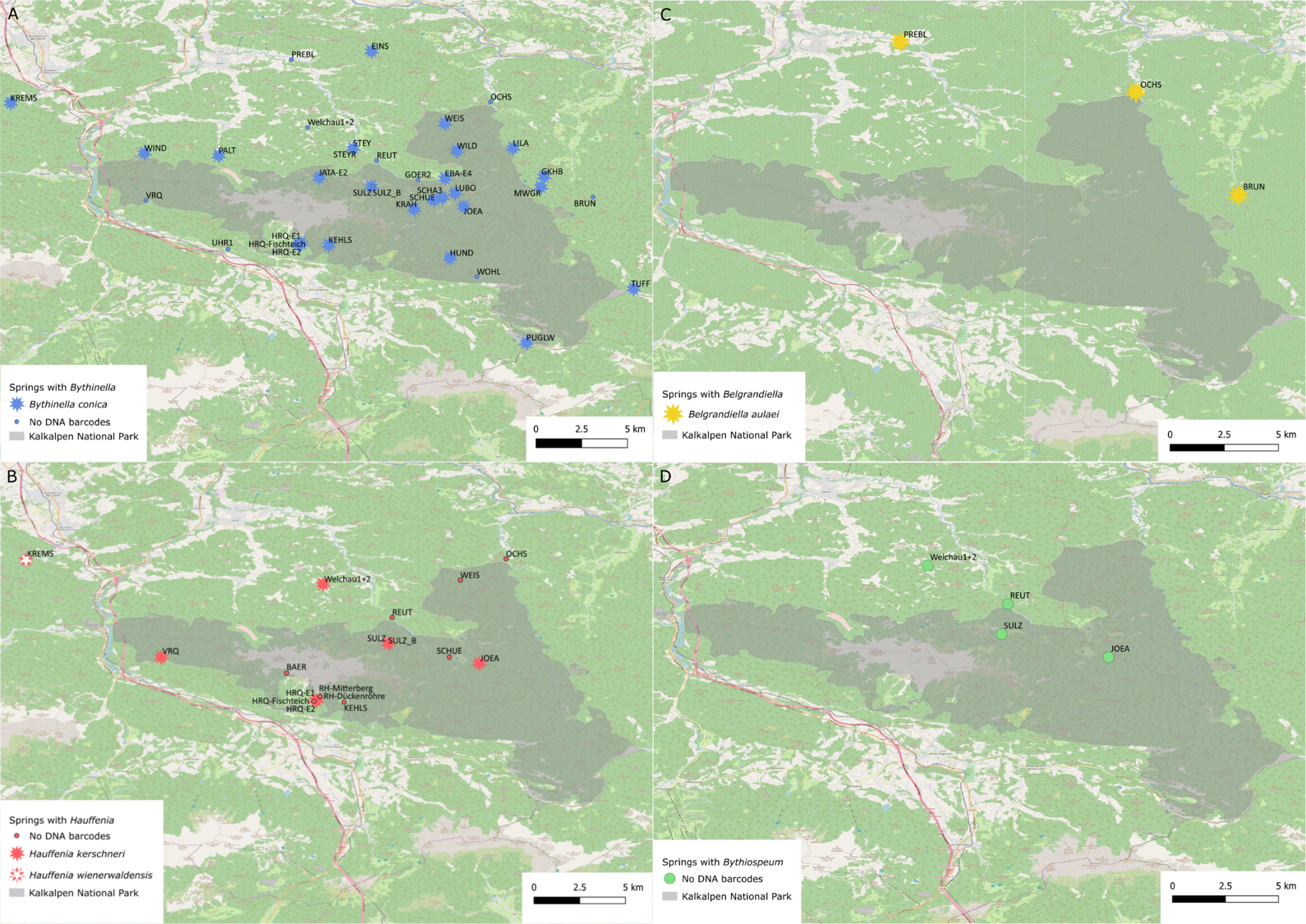

Supplement: Supplementary material 4 — Supporting Figure 2 [file bdj-11-e91496-s004.png]
